# The allosterically modulated FFAR2 is transactivated by signals generated by other neutrophil GPCRs

**DOI:** 10.1371/journal.pone.0268363

**Published:** 2023-04-06

**Authors:** Simon Lind, Kenneth L. Granberg, Huamei Forsman, Claes Dahlgren

**Affiliations:** 1 Department of Rheumatology and Inflammation Research, Institute of Medicine, Sahlgrenska Academy, University of Gothenburg, Gothenburg, Sweden; 2 Medicinal Chemistry, Research and Early Development, Cardiovascular, Renal and Metabolism, BioPharmaceuticals R&D, AstraZeneca, Gothenburg, Sweden; Weizmann Institute of Science, ISRAEL

## Abstract

Positive allosteric modulators for free fatty acid receptor 2 (FFAR2/GPR43), that affect receptor function through binding to two distinct allosteric binding sites, were used to determine the correlation between the responses induced in neutrophils by two distinct activation modes; FFAR2 was activated either by the orthosteric agonist propionate or by a receptor transactivation mechanism that activated FFAR2 from the cytosolic side of the neutrophil plasma membrane by signals generated by the neutrophil PAFR (receptor for platelet activating factor), P2Y_2_R (receptor for ATP), FPR1 (receptor for fMLF) and FPR2 (receptor for WKYMVM). We show that the transactivation signals that activate FFAR2 in the absence of any orthosteric agonist were generated downstream of the signaling G protein that couple to PAFR and P2Y_2_R. This transactivation of allosterically modulated FFAR2s, by signals generated by PAFR/P2Y_2_R, represents a novel mechanism by which a G protein coupled receptor can be activated. Weak correlations were obtained when the FFAR2 activity was induced by the transactivation signals generated by PAFRs and P2Y_2_Rs were compared with the FFAR2 activity induced by the orthosteric agonist propionate. Comparison of the responses for each allosteric modulator revealed that the ratio values, calculated from the peak values of the ATP and propionate responses, varied from 0.2 to 1. Depending on the allosteric modulator, the response induced by the two different mechanisms (orthosteric activation and receptor transactivation, respectively), was equal or the propionate response was more pronounced. Importantly, we conclude that FFAR2 activation from outside (orthosteric activation) and inside (receptor cross-talk/transactivation) can be selectively affected by an allosteric FFAR2 modulator.

## 1. Introduction

The short chain free fatty acid receptor 2 (FFAR2/GPR43) is a member of the large family of G protein coupled receptors (GPCRs; also known as 7-transmembrane receptors, 7TMR) expressed in neutrophils and the receptor has been suggested to have important roles in the regulation of inflammation [1, 2]. The GPCRs have a common basic structure with a peptide chain spanning the membrane seven times and these receptors constitute the basis for how cells recognize specific signaling molecules in their environment [3, 4]. Generally, parts of the receptor exposed on the cell surface specifically recognize agonists, while cytosolic parts of the receptor initiate/transfer the agonist induced signaling events that regulate a large number of biological processes down-stream of the activated receptor [5–7]. The agonist-interaction and receptor signaling scheme was for long assumed to be some type of on/off switch, but this has during recent years been shown to be an oversimplification. This is illustrated not only by the fact that some agonists are biased and trigger the recognizing receptor to preferentially activate one signal-transduction pathway over another, but also by the fact that many (if not all) receptors have regulatory (allosteric) binding sites that are both structurally and physically separated from the orthosteric binding sites [6, 8]. Such allosteric binding sites recognize non-orthosteric ligands (modulators) that change the receptor activities induced by orthosteric agonists. Propionate is an orthosteric FFAR2 agonists, but the activity of the FFAR2 can also be modulated by non-activating ligands binding to allosteric sites [9–11]. Accordingly, allosteric FFAR2 modulators transfer the natural low‐activating orthosteric agonist propionate to a potent activating ligand. The original paradigm for GPCR down-stream signaling stated that a receptor specific agonist activates only one particular receptor and a receptor specific allosteric modulator affects only signaling induced by orthosteric agonists that are recognized by the modulated receptor [12]. However, these limitations have been challenged and activation is now known to be triggered both by promiscuous agonists that are recognized by more than one receptor [13], as well as by receptor transactivation/cross-talk signals that activate an allosterically modulated receptor without the involvement of any orthosteric agonist that is recognized by the modulated receptor [14]. In neutrophils, this type of receptor cross-talk activation/transactivation was originally shown to be a way to transfer desensitized receptors from a non-signaling to a signaling state. This process has been extensively studied in neutrophils with desensitized Formyl Peptide Receptors (FPRs) that are reactivated/transactivated by cross-talk signals generated by other pattern recognition neutrophil GPCRs [15–18]. The identity of the signals that transactivate desensitized FPRs have not yet been identified, but the results obtained suggest that the transactivation signals are generated on the cytosolic side of the membrane, down-stream of the G protein coupled to the transactivating receptor partner (described in more detail in [13]).

We have previously shown that the positive allosteric FFAR2 modulators Cmp58 and AZ1729 are recognized by two distinct different allosteric receptor sites [8, 9]. These modulators transfer not only propionate to a potent neutrophil activating agonist, but also the responses induced by ATP (an agonist specific for P2Y_2_R) and the two formyl peptide receptors (FPR1 and FPR2) [10, 11, 19]. The mechanism for the receptor transactivation of the allosterically modulated FFAR2 has notable similarities with the receptor cross-talk/transactivation of desensitized FPRs [13, 20]. This is illustrated by the fact that, i) the ATP/P2Y_2_R mediated response inhibited by FFAR2 antagonists and, ii) the ATP induced transactivation of FFAR2 is abolished by an inhibition of Gα_q_ subunit coupled to P2Y_2_R [10, 11, 19]. This inhibition is achieved despite the fact that the Gα_q_ inhibitor lacks direct effects on FFA2R-mediated responses [10, 11, 19]. The Gα_q_ dependency of the P2Y_2_R is thus, transferred to FFA2R and the crosstalk signals that transactivate this receptor are generated downstream of the Gα_q_ containing G protein coupled to P2Y_2_R. Taken together, these observations suggest that the allosterically modulated FFAR2s are transactivated from inside the plasma membrane, by not yet defined signals generated down-stream of the G protein coupled to P2Y_2_R [10, 19].

We recently characterized several structurally diverse compounds that, based on their positive modulating effects on the neutrophil response induced by propionate, were classified as allosteric FFAR2 modulators [9]. These allosteric modulators interact with either of two distinctly different allosteric modulation sites in FFAR2 and we herein used these as molecular tools to determine the link (if any) between activation by the orthosteric agonist propionate and by the receptor transactivation signals generated on the inside of the plasma by cross-talking GPCRs. We show that in addition to the agonist/receptor pairs (i.e., ATP/P2Y_2_R, fMLF/FPR1 and WKYMVM/FPR2) earlier shown to transactivate FFAR2, also the receptor for platelet activating factor (PAFR) generates signals that transactivate FFAR2. When comparing transactivation of FFAR2 mediated by the Gα_i_ coupled FPR1 and FPR2, the activation patterns were very similar to those mediated by the P2Y_2_R, FPR1 and FPR2. Although not as conclusive, there were also similarities between how the allosteric modulators affect the Gα_q_ coupled PAF and ATP induced neutrophil NADPH oxidase activity. More importantly, the positive allosteric effect was selective for propionate suggesting that the mechanisms that regulate activation of the receptor from outside (orthosteric activation) and inside (cross-talk/transactivation) are different.

## 2. Material and methods

### 2.1. Chemicals

Isoluminol, TNF-α, fMLF, ATP, PAF and propionic acid, were purchased from Sigma (Sigma Chemical Co., St. Louis, MO, USA). Dextran and Ficoll-Paque were obtained from GE-Healthcare Bio-Science (Uppsala, Sweden). Horseradish peroxidase (HRP) was obtained from Boehringer Mannheim (Mannheim, Germany). The PAF agonist were from Avanti Polar Lipids Inc. (Alabama, USA), and all transactivating receptor agonist tools are described in Table 1. The allosteric FFAR2 modulator Cmp58 ((*S*)-2-(4-chlorophenyl)-3,3-dimethyl-*N*-(5-phenylthiazol-2-yl)butanamide, the FFAR2 antagonists GLPG0974 (4-[[(*R*)-1-(benzo[*b*]thiophene-3-carbonyl)-2-methyl-azetidine-2-carbonyl]-(3-chlorobenzyl)amino]-butyric acid) and AR-C118925 {5-[[5-(2,8-dimethyl-5*H*-dibenzo[*a*,*d*]cyclohepten-5-yl)-3,4-dihydro-2-oxo-4-thioxo-1(*2H*)-pyrimidinyl]methyl]-*N*-*2H*-tetrazol-5-yl-2-furancarboxamide} were obtained from Calbiochem-Merck Millipore (Billerica, USA) and TOCRIS (Bristol, UK). The antagonist CATPB ((*S*)-3-(2-(3-chlorophenyl)acetamido)-4-(4-(trifluoromethyl)phenyl) butanoic acid was synthesized as described previously [21, 22] and obtained (as generous gifts) from Trond Ulven (Odense University, Denmark). The Gα_q_ inhibitor YM-254890 was purchased from Wako Chemicals (Neuss, Germany). The FPR2 specific hexapeptide Trp-Tyr-Met-Val-Met-NH2 (WKYMVM) was synthesized and purified by HPLC by Alta Bioscience (University of Birmingham, Birmingham, United Kingdom). The allosteric FFAR2 modulator AZ1729 [9, 11, 23] together with all the other FFAR2 ligands included in the study (Fig 2) were provided by AstraZeneca (Gothenburg, Sweden).

**Table 1:** Classification of FFAR2 ligands in neutrophils.

Compound	Activation^†^	Allosteric modulation of			Classification
		Propionate	AZ1729	Cmp58	
Cmp58	-	+	+	-	Cmp58 AM*
AZ5994	+	+	+	-	Cmp58-like agonist
AZ7004	-	+	+	-	Cmp58-like AM
CATPB	-	-	-	-	Antagonist
GLPG0974	-	-	-	-	Antagonist
AZ1729	-	+	-	+	AZ1729 AM
AZ0688	-	+	-	+	AZ1729-like AM
AZ0682	-	+	-	+	AZ1729-like AM
AZ8703	-	+	-	+	AZ1729-like AM
AZ1702	-	+	-	+	AZ1729-like AM

^†^ Indicates agonistic properties in neutrophils

*AM: Allosteric modulator

Subsequent dilutions of receptor ligand and other reagents were made in Krebs-Ringer Glucose phosphate buffer (KRG; 120 mM NaCl, 4.9 mM KCl, 1.7 mM KH_2_PO_4_, 8.3 mM NaH_2_PO_4_, 1.2 mM MgSO_4_, 10 mM glucose, and 1 mM CaCl_2_ in dH_2_O, pH 7.3).

### 2.2. Isolation of human neutrophils

Neutrophils were isolated from buffy coats from healthy blood donors by dextran sedimentation and Ficoll-Paque gradient centrifugation as described by Bøyum [24]. Remaining erythrocytes were removed by hypotonic lysis and the neutrophils were washed and resuspended in KRG. More than 90% of the cells were neutrophils with a viability of >95%. We have earlier shown that there are no qualitative differences in the response induced in naïve and TNFα primed neutrophils [25, 26] but the responses obtained with the primed cells are substantially higher and by that easier to quantify/analyze. Accordingly, the activation signals were amplified by TNF-α priming. The neutrophils used were first primed with TNF-α (10 ng/mL for 20 min at 37°C), and then stored on ice until use.

### 2.3. Measuring NADPH-oxidase activity

Isoluminol-enhanced chemiluminescence (CL) technique was used to measure superoxide production [27], the precursor of production of reactive oxygen species (ROS), by the NADPH-oxidase activity as described [26, 28]. In short, the measurements were performed in a six-channel Biolumat LB 9505 (Berthold Co., Wildbad, Germany), using disposable 4 mL polypropylene tubes and a 900 μL reaction mixture containing 10^5^ neutrophils, isoluminol (0.2 μM) and HRP (4 Units/mL). The tubes were equilibrated for 5 min at 37°C, before addition of agonist (100 μL) and the light emission was recorded continuously over time. In experiments where the effects of receptor specific antagonists were determined, these were added to the reaction mixture 1–5 min before stimulation and control neutrophils were separately incubated under the same conditions but in the absence of an antagonist.

### 2.4. Statistical analysis

Statistical calculations were performed in GraphPad Prism 9.3 (Graphpad Software, San Diego, CA, USA). Which specific statistical tests that are performed are stated in the relevant figure legend. A *p*‐value < 0.05 was regarded as statistically significant difference and is indicated by *p < 0.05, **p < 0.01, ***p < 0.001. Correlation between the different modes of activation was performed and presented with Pearson coefficients and plotted together with simple linear regression showing 95% confidence intervals. To further study the agreement a Bland Altman plot [29] was calculated showing bias and the limits of the agreement. Some data was also further statistically analyzed using a one-way ANOVA followed by Dunnett’s multiple comparison or, paired Student’s *t*-test. Even though the results in the figures are presented as percent of control values, the statistics have been calculated using the raw data.

### 2.5. Ethics statement

In this study, conducted at the Sahlgrenska Academy in Sweden, buffy coats obtained from the blood bank at Sahlgrenska University Hospital, Gothenburg, Sweden have been used. According to the Swedish legislation section code 4§ 3p SFS 2003:460 (Lag om etikprövning av forskning som avser människor), no ethical approval was needed since the buffy coats were provided anonymously and could not be traced back to a specific donor.

## 3. Results

### 3.1. Two distinct different mechanisms used by FFAR2 to activate the neutrophil NADPH-oxidase

#### 3.1.1. Activation by the orthosteric FFAR2 agonist propionate (Fig 1)

In order for propionate, an orthosteric FFAR2 agonist, to activate the neutrophil electron transporting NADPH-oxidase system that produces superoxide anions (O_2_^-^), the receptor must be allosterically modulated (see [9] and [30]). In agreement with the receptor-selectivity of propionate, the response induced by this agonist in the presence of either of the two allosteric modulators Cmp58 and AZ1729 (Fig 2), was inhibited by an FFAR2 antagonist; for comparison the response was not affected by an antagonist specific for P2Y_2_R, the neutrophil receptor for ATP (Fig 3A and 3B). The allosteric modulators Cmp58 and AZ1729 have been shown to be specific for FFAR2 [23, 31] and to be recognized by two different allosteric receptor sites [9, 11]. The response induced by propionate was not affected by the Gα_q_ inhibitor YM-254890 (Fig 3B), showing that no Gα_q_ containing G protein is involved in the down-stream signaling of propionate activated FFAR2s.

**Fig 1:**
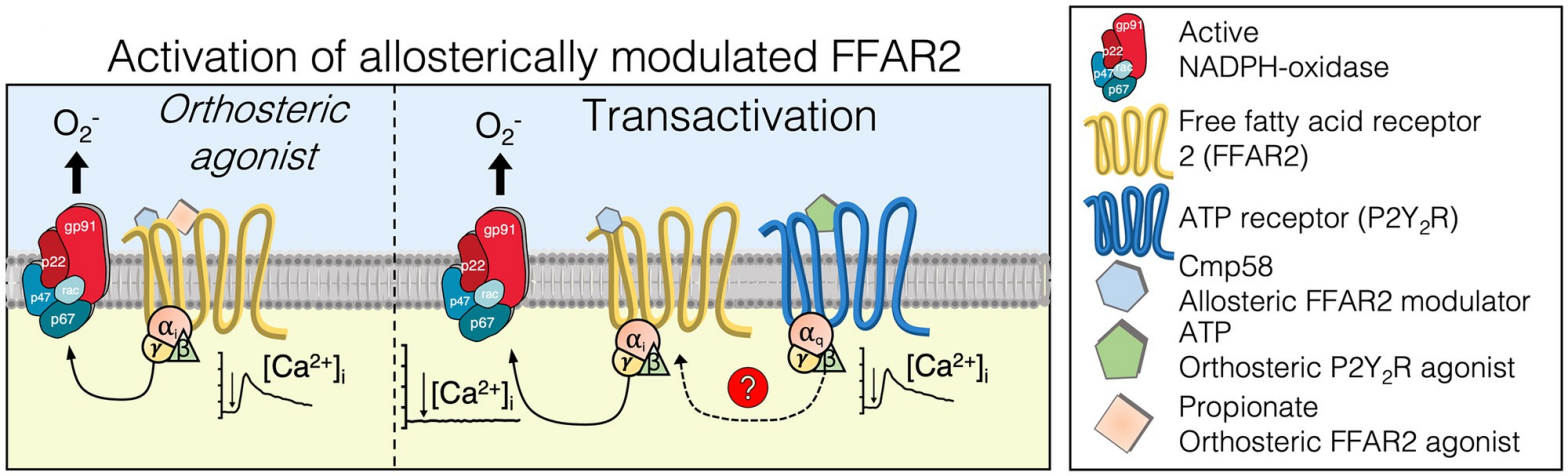
Activation of allosterically modulated FFAR2 by two different mechanisms. **Left**: Activation of FFAR2 by the orthosteric agonist propionate includes a transient rise in [Ca^2+^]_i_ and production of O_2_^-^. **Right**: Transactivation of FFAR2 by signals generated by the ATP receptor (inducing a rise in [Ca^2+^]_i_ by itself) leads to production of O_2_^-^ but no FFAR2 induced transient rise in [Ca^2+^]_i_.

**Fig 2:**
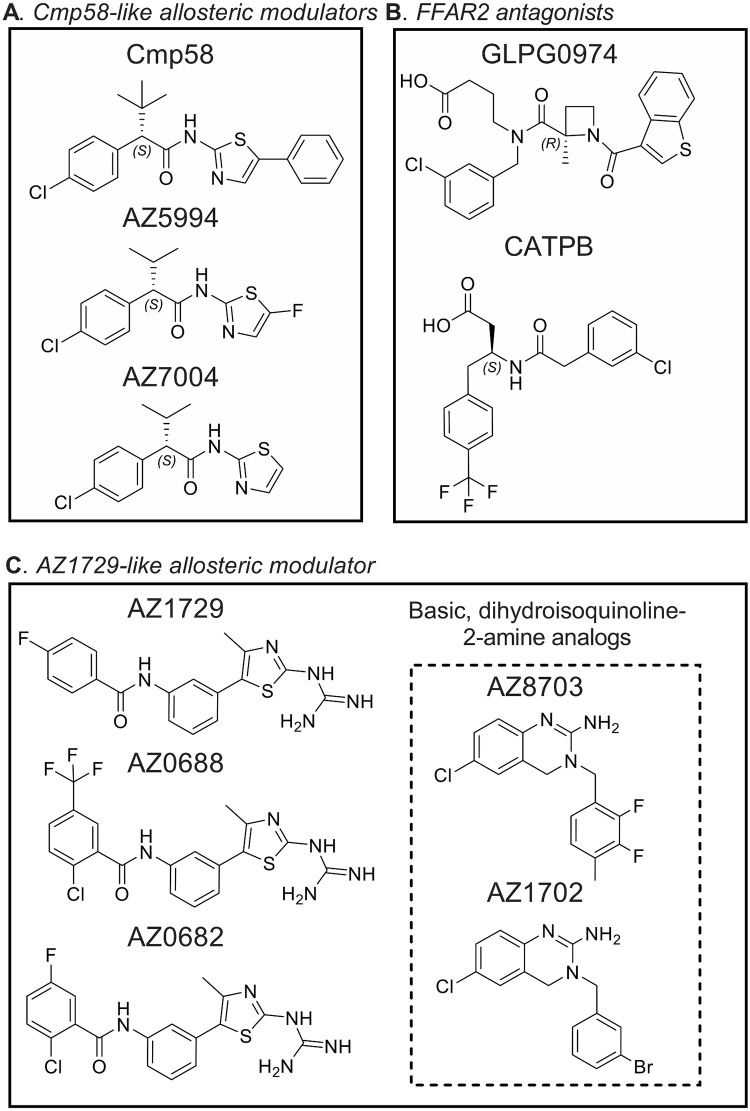
Chemical structures of the FFAR2 ligands used in the study. Structural variants of allosteric FFAR2 modulators with distinct interaction properties were chosen through screening of a mini-library of small compounds. Ten compounds were identified as allosteric FFAR2 modulators. Two selective FFAR2 antagonists, GPLG0974 and CATPB, were also selected.

**Fig 3:**
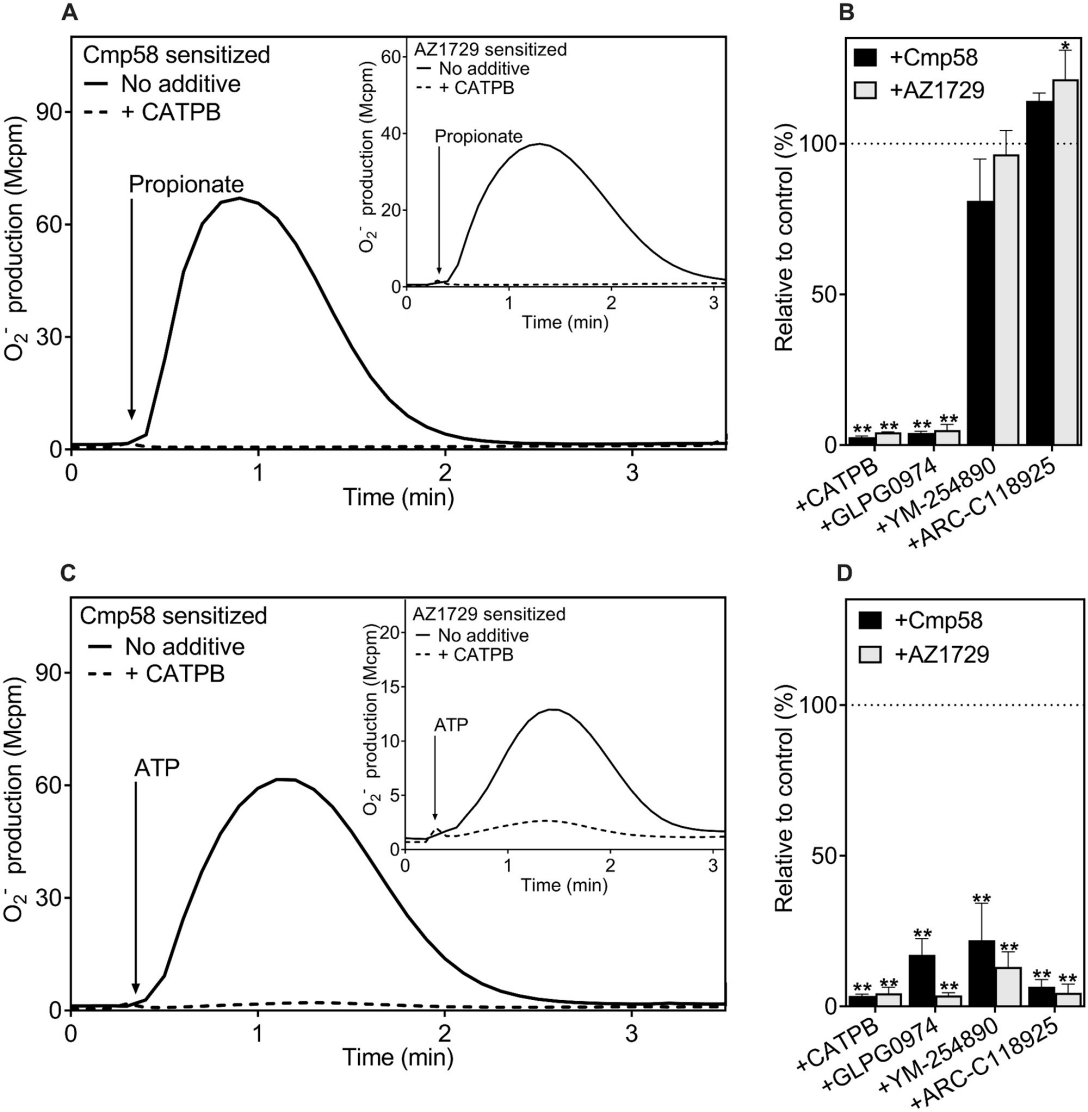
Selective inhibition by specific antagonists/inhibitors on the neutrophil FFAR2 dependent response when triggered by the orthosteric FFAR2 agonist propionate and the P2Y2R agonist ATP, respectively. (**A**) Neutrophils sensitized with Cmp58 (1 μM for 5 min) were activated (indicated with arrow) with the FFAR2 orthosteric agonist, propionate (25 μM solid line). The release of O_2_^-^ was measured continuously and expressed in Mcpm. The response induced by propionate in Cmp58 sensitized (1 μM for 5 min) neutrophils was also treated at the same time with the FFAR2 specific antagonists, CATPB (dashed line, 100 nM for 5 min). One of four representative experiment is shown. Inset: Same experimental setup was used but neutrophils were instead sensitized to FFAR2 specific allosteric modulator, AZ1729 (1 μM for 5 min). (**B**) The peak O_2_^-^ production values were determined and the ratios between the propionate modulated responses from Cmp58 (1 μM) or AZ1729 (1 μM) in the absence and presence of one the FFAR2 antagonist CATPB (100 nM) and GLPG0974 (100 nM), the Gα_q_ inhibitor YM-254890 (200 nM), the selective P2Y_2_R antagonist AR-C118925 (1 μM) were calculated and expressed in remaining activity (in percent) in the presence of the respective antagonist (mean + SD, n = 4). (**C, inset**) Same experimental setup as in (**A**) but the activating ligand is instead the P2Y_2_ agonist, ATP (10 μM). (**D**) The peak O_2_^-^ production values were determined and the ratios between the responses in the ATP modulated responses from Cmp58 (1 μM) or AZ1729 (1 μM) in absence and presence of the respective antagonist CATPB (100 nM) or GLPG0974 (100 nM), YM-254890 (200 nM), and AR-C118925 (1 μM) were calculated and expressed in remaining activity (in percent) in the presence of the respective antagonist (mean+SD, n = 4). Statistical analyses were performed (**B** and **D**) using a one-way ANOVA followed by a Dunnett’s multiple comparison test comparing the peak responses in the absence and presence of respective inhibitor.

#### 3.1.2. Activation by ATP, an orthosteric P2Y_2_R agonist (Fig 1)

No activation of the O_2_^-^ producing NADPH-oxidase was induced by the P2Y_2_R agonist ATP in naive neutrophils (earlier shown in [10, 11, 19]). In the presence of either of the allosteric FFAR2 modulators Cmp58 or AZ1729, ATP is a potent activating agonist (Fig 3C). The differences in the inhibition patterns of a selection of inhibitors/antagonists show that the orthosteric FFAR2 agonist propionate and the P2Y_2_R agonist ATP trigger different pathways to activate FFAR2. The ATP induced response was in contrast to the propionate induced response, inhibited both by the P2Y_2_R specific antagonist AR-C118925 and by the Gα_q_ selective inhibitor YM-254890 (Fig 3D). In addition, and irrespectively of the allosteric FFAR2 modulator included, also the ATP induced response was fully inhibited by the FFAR2 specific antagonists CATPB and GLPG0974 (Fig 3C and 3D). The effect of the allosteric modulators on the propionate-induced response is there also when the order by which the two ligands are added to the cells is reversed and the orthosteric agonist is used as a sensitizing (priming) ligand. This is in accordance with the defined characteristics of an allosteric modulator that should be reciprocal in nature. The novel transactivation mechanism effect is not reciprocal [20]. Taken together, these data show that an activation of allosterically modulated FFAR2s can be achieved by two different mechanisms induced by a conventional orthosteric FFAR2 agonist independent of Gα_q_ and by the P2Y_2_R agonist, by signals generated down-stream of a Gα_q_ containing G protein, respectively.

### 3.2. Ligand tools for FFAR2

We have earlier shown that FFAR2 has two different allosteric binding site [11], and the modulaters used in this study were classified as “Cmp58 like” (activation achieved only together with AZ1729) or as “AZ1729 like” (activation achieved only together with Cmp58) (Table 2). Allosteric FFAR2 modulators (in total eight; Fig 2) were used as tools to determine differences (if any) related to the activation mechanism by which the allosteric modulated FFAR2s were activated by the two different mechanisms described above.

**Table 2:** Classification of orthosteric agonists and their respective GPCR. The neutrophil interaction characteristic of propionate and the receptor specific ligands recognized by P2Y_2_R (i.e., ATP), PAFR (i.e., PAF) and FPRs (i.e., the peptides fMLF and WKYMVM) used in the study have earlier been described in more details (see the references [16, 17, 44, 45]) and it is important to point out that with the concentrations used, the agonists alone (without the allosteric FFAR2 modulators) were unable to activate the NADPH-oxidase.

Agonist	Recognizing receptor (GPCR)
Propionate	Free Fatty Acid Receptor 2 (FFAR2)
ATP	P2Y purinoceptor 2 (P2Y_2_R)
PAF	Platelet Activating Factor Receptor (PAFR)
fMLF	Formyl Peptide Receptor 1 (FPR1)
WKYMVM	Formyl Peptide Receptor 2 (FPR2)

### 3.3. Relation between the neutrophil responses when FFAR2 was activated by two different activation mechanisms

#### 3.3.1. Correlation between the FFAR2 transduced neutrophil responses induced by propionate and ATP

Previous studies [10, 19] and Fig 3 show that the specific P2Y_2_R agonist ATP as well as the specific FFAR2 agonist propionate activate FFAR2 provided that the receptor is allosterically modulated. As described above, however, the activation mechanisms differ. The eight allosteric FFAR2 modulators (Table 2) were used to determine the direct relation between the neutrophil responses when FFAR2 was activated by the “propionate and ATP mechanism”, respectively. The allosteric FFAR2 modulating compounds were allowed to sensitize neutrophils during a 5 min incubation with respective compound. The neutrophils were then activated with propionate (25 μM) or ATP (10 μM) and the production of superoxide (O_2_^-^) was followed over time. The peak values of the responses were determined, and eight independent experiments were performed.

**Fig 4:**
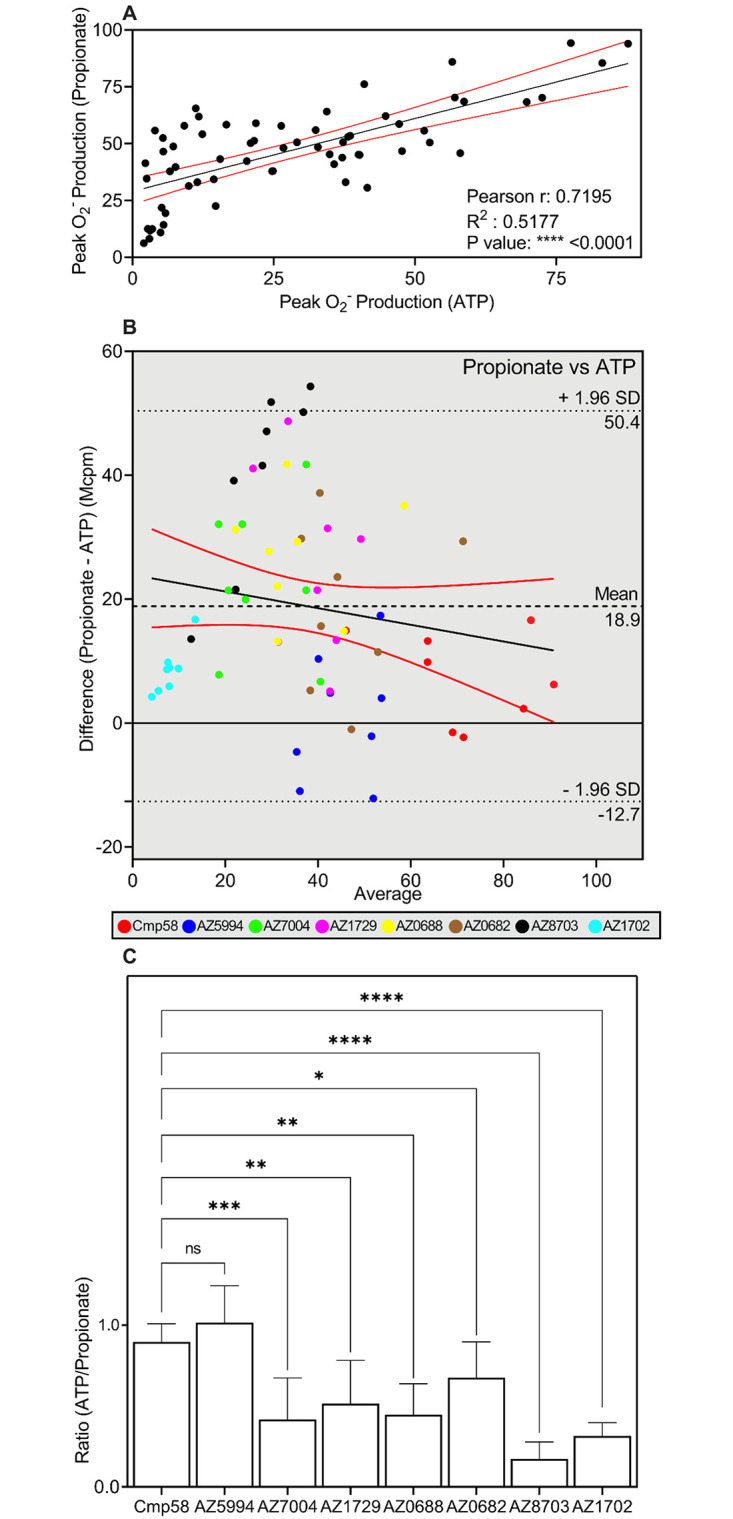
Activation by propionate or ATP of neutrophils sensitized with allosteric FFAR2 modulators. (**A**-**C**) Human neutrophils were sensitized for 5 min with allosteric FFAR2 modulators (1 μM each) and activated either by propionate (25 μM) or ATP (10 μM). O_2_^-^ production was measured continuously and the peak responses in neutrophils activated with propionate and ATP, respectively were determined for each allosteric modulator. Eight independent experiments were performed with cells isolated from healthy donors. (**A**) Scatter plot for correlation between the peak values (O_2_^-^ production in relative light units; Mcpm) with propionate (25 μM; ordinate) and ATP (10 μM; abscissa) as the activating agonists. The responses obtained with all allosteric FFAR2 modulators were included. The black solid line corresponds to a linear regression fit and the red solid lines correspond to the 95% confidence slope for the linear regression fit. Pearson coefficients are also given. (**B**) Bland-Altman analysis of the peak values (O_2_^-^ production in relative light units; Mcpm) with propionate and ATP as the activating agonists. The responses obtained with all allosteric FFAR2 modulators were included and used to estimate the agreement between the readouts. The Bland-Altman plot for the differences (values for propionate–values for ATP in Mcpm) were plotted (ordinate) against (abscissa) the averages (peak values of (propionate + ATP)/2). Line of equality is presented (black dashed line), and the 95% upper and lower CI of limits of agreement are included (black dotted lines). Linear regression line of the differences is drawn as a black line that is combined with the 95% confidence interval (red lines above and below the linear regression line. The responses induced by propionate and ATP, respectively, have been color-coded with a color that is unique for each allosteric FFAR2 modulator included in the Bland-Altman plot. (**C**) The ratio for each allosteric FFAR2 modulator, between the PAF and ATP responses (O_2_^-^ production in relative light units; Mcpm) were calculated and presented in a bar graph. One-way ANOVA followed by Dunnett’s post-hoc test was used to calculate significant difference deviation from the ratio obtained with Cmp58 as the amplifying allosteric FFAR2 modulator. Eight independent experiments with cells isolated from healthy donors were performed and the results are given as mean +SD.

There are multiple ways to determine association between two responses. A direct application of the correlation coefficient (*r*) was used to determine the direction (positive or negative) and quantify the strength of the association (if any) between the propionate and ATP induced responses. Accordingly, to determine the presumable linear correlation between responses of allosterically modulated FFAR2s when triggered by propionate and ATP, respectively, a scatter plot was generated followed by a linear regression curve as well as a Pearson correlation calculation of r and R^2^) was performed. The peak response values from all experiments performed with the different allosteric modulators were included in the plot (Fig 4A). The linear regression and the Pearson correlation value (r ≈ 0.72), supports a moderate to strong positive linear correlation between the two data sets. A portion of data points were, however, not in the area that defined the 95% confidence interval (marked by red lines in Fig 4A). The R^2^ ≈ 0.52 means that approximately half of the observed variation could be explained by the model.

#### 3.3.2. Agreement between the FFAR2 transduced responses induced by propionate and ATP analyzed by the Bland-Altman technique

An alternative approach to the conventional method to evaluate the level of agreement between two data sets was introduced by Altman and Bland in 1986 [29, 32], and this method has become frequently used as a suitable analytical tool to determining the limits of agreement between data sets obtained with two different quantitative methods [29, 32]. Also, the Bland-Altman estimation of agreement is based on a scatter data plot in which the values of the differences between paired measurements (A-B = Y value) are plotted against the mean values of the two [(A+B)/2 = X value]. Accordingly, using this equation in the Bland-Altman graph describing the effects of the different allosteric modulators, the Y values in the plot equals the propionate induced neutrophil response (peak value of O_2_^-^ production (A)) from which the corresponding ATP induced response (B) was subtracted, and the X values equals the mean value of the propionate response and the corresponding ATP response (Fig 4B). This graph provides two main pieces of information; i) the average of all the differences that was 18.9 units and ii) the 95% limits of agreement with a lower limit of -12.7 and an upper of 50.4 units. A full support for the significant positive correlation suggested by the Pearson r values (Fig 4A) would require that the data points in the Bland-Altman plot lie perfectly along the line of equality. The data show that compared to ATP, the propionate response measures on the average an 18.9 Mcpm higher value. It is recommended that 95% of the data points should lie within ±1.96 SD of the mean difference–limits of agreement, and only two data points exceeds 50.4 Mcpm, which indicates there are agreement between the tests. Applying a simple linear correlation with confidence interval limits plot to look for proportional differences [33, 34] show that there is a slight negative bias. Even though most values fall within the limits of agreement, the different allosteric modulators clustered in different parts of the Bland-Altman plot (Fig 4B). This suggests that although there is an overall positive correlation there is not a direct agreement between the two responses for different modulators.

#### 3.3.3. The allosteric FFAR2 modulators affect the propionate and ATP responses differently

A simpler and more direct way to determine the similarities/differences between the effects of the allosteric FFAR2 modulators on the NADPH-oxidase activity induced by propionate and ATP, is to compare the two responses separately for each allosteric modulator (shown in Fig 4C). The ratio between the peak values of the ATP and propionate responses was close to one with Cmp58 as well as AZ5994 whereas the values for the rest of the allosteric FFAR2 modulators were lower and significantly different when compared to Cmp58 (Fig 4C). Taken together, the data presented suggest that the allosteric modulators affect the propionate and ATP induced responses differently.

#### 3.3.4. Relation between the neutrophil responses when FFAR2 was activated through receptor cross-talk by two Gα_q_ coupled GPCRs

**Fig 5:**
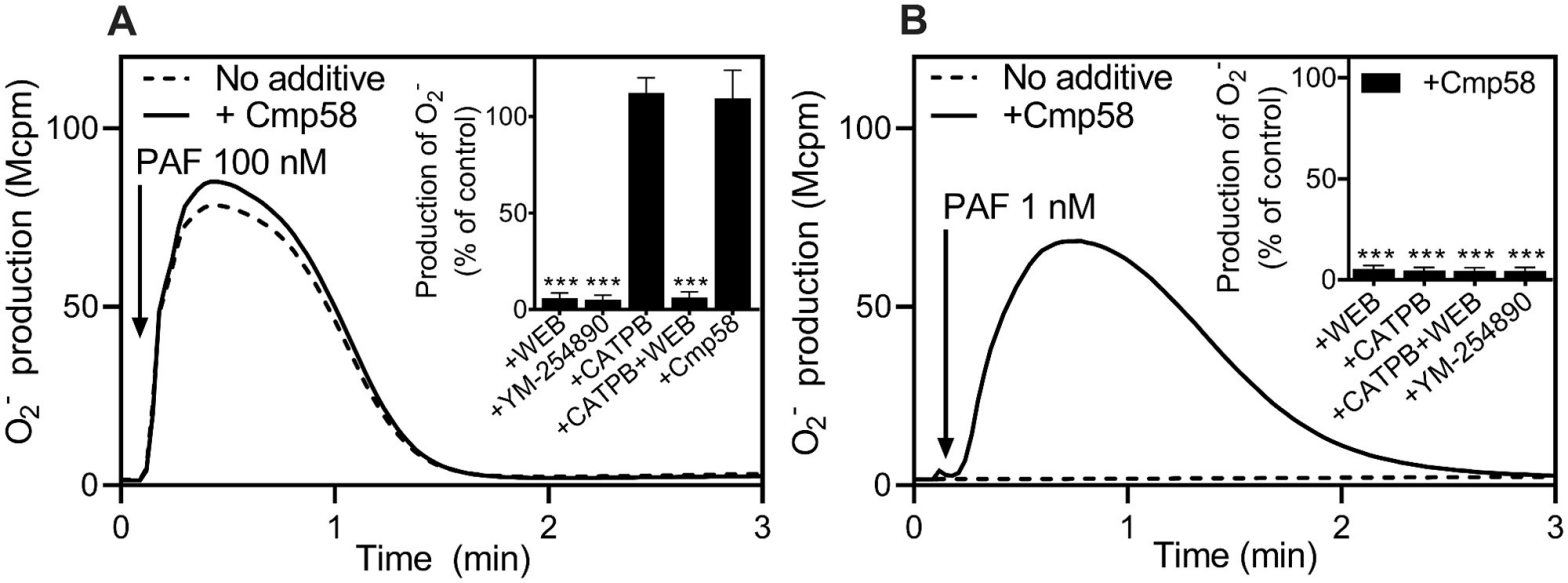
Selective inhibition by specific antagonists/inhibitors on the neutrophil FFAR2 dependent cross-talk response when triggered by the orthosteric PAFR agonist PAF. (**A**) Neutrophils sensitized with Cmp58 (1 μM for 5 min solid line) or without (dashed line) were activated (indicated with arrow) with the PAFR orthosteric agonist, PAF (100 nM). The release of O_2_^-^ was measured continuously and expressed as Mcpm. One representative experiment out of three is shown. (**A,** inset) The PAF response was also treated at the same time by the PAFR specific antagonist WEB (1 μM), Gα_q_ specific inhibitor YM-254890 (200 nM), FFAR2 specific antagonists, CATPB (100 nM for 5 min) as well as the combination of CAPTB (100 nM) and WEB (1 μM). The peak O_2_^-^ production values were determined and the ratios between the PAF responses in the absence and presence of the respective were calculated and expressed in remaining activity (in percent) in the presence of the respective antagonist (mean + SD, n = 3). (**B**) An experimental setup as that in **A** was used but with a 100-fold lower concentration of PAF (1 nM). (**B, inset**) Similar to (**A, inset**), however the peak O_2_^-^ production values were determined and the ratios between the responses in the PAF (1 nM) modulated responses from Cmp58 (1 μM in absence and presence of the respective antagonist/inhibitor.

*The allosterically modulated FFAR2 is transactivated by the receptor for platelet activating factor (PAFR)*. Binding of the lipid neutrophil chemoattractant PAF to its neutrophil receptor triggers an activation of the neutrophil NADPH-oxidase, and similar to P2Y_2_R the receptor down-stream signaling by the PAFR relies on a Gα_q_ containing G protein (Fig 5A). Depending on the concentration of PAF, Cmp58 was without effect (100 nM concentration of PAF; Fig 5A) or turned a non-activating concentration (1 nM; Fig 5B) into a potent transactivating ligand. The PAF induced transactivation was inhibited not only by the PAFR specific antagonist WEB and by the Gα_q_ selective inhibitor YM-254890 but also by the FFAR2 specific antagonists CATPB. Taken together, these data show that PAF induced activation of allosterically modulated FFAR2s was achieved by a receptor cross-talk/transactivation mechanism, similar to that induced by ATP (see above).

Statistical analyses were performed (**A** and **B, insets**) using a one-way ANOVA followed by a Dunnett’s multiple comparison test comparing the peak PAF responses in the absence and presence of respective treatment.

**Fig 6:**
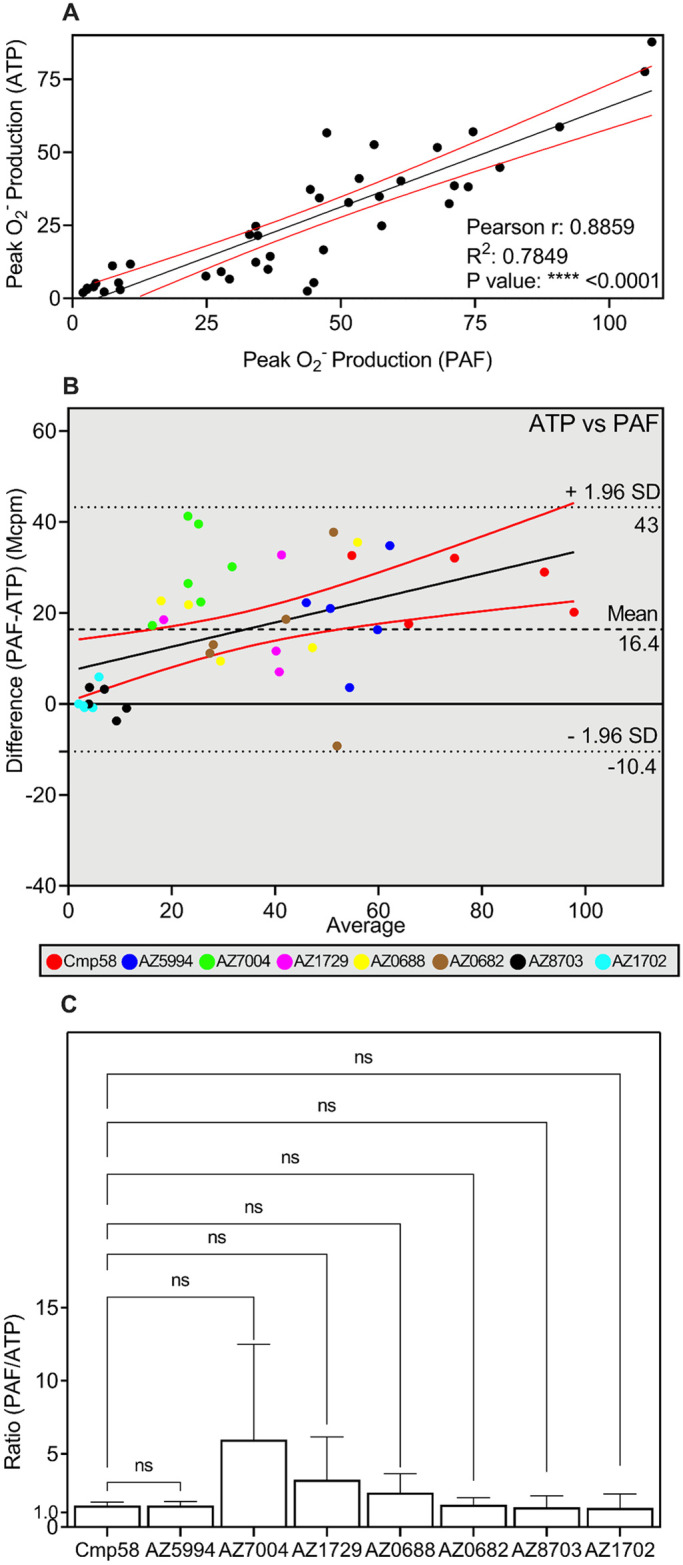
Activation by PAF or ATP of neutrophils sensitized with allosteric FFAR2 modulators. (**A**-**C**) Human neutrophils were sensitized for 5 min with allosteric FFAR2 modulators (1 μM each) and activated either by PAF (1 nM) or ATP (10 μM). O_2_^-^ production was measured continuously and the peak responses in neutrophils activated with propionate and ATP, respectively were determined for each allosteric modulator. Six independent experiments were performed with cells isolated from healthy donors. (**A**) Scatter plot for correlation between the peak values (O_2_^-^ production in relative light units; Mcpm) with PAF (1 nM; abscissa) and ATP (10 μM; ordinate) as the activating agonists. The responses obtained with all allosteric FFAR2 modulators were included. The black solid line corresponds to a linear regression fit and the red solid lines correspond to the 95% confidence slope for the linear regression fit. Pearson coefficients are also given. (**B**) Bland-Altman analysis of the peak values (O_2_^-^ production in relative light units; Mcpm) with PAF and ATP as the activating agonists. The responses obtained with all allosteric FFAR2 modulators were included and used to estimate the agreement between the readouts. The Bland-Altman plot for the differences (values for PAF–values for ATP in Mcpm) were plotted (ordinate) against (abscissa) the averages (peak values of (PAF + ATP)/2). Line of equality is presented (black dashed line), and the 95% upper and lower CI of limits of agreement are included (black dotted lines). Linear regression line of the differences is drawn as a black line that is combined with the 95% confidence interval (red lines above and below the linear regression line. The responses induced by propionate and ATP, respectively, have been color-coded with a color that is unique for each allosteric FFAR2 modulator included in the Bland-Altman plot. (**C**) The ratio for each allosteric FFAR2 modulator, between the PAF and ATP responses (O_2_^-^ production in relative light units; Mcpm) were calculated and presented in a bar graph. One-way ANOVA followed by Dunnett’s post-hoc test was used to calculate significant difference deviation from the ratio obtained with Cmp58 as the amplifying allosteric FFAR2 modulator. Six independent experiments with cells isolated from healthy donors were performed and the results are given as mean +SD.

*Correlation between the FFAR2 transduced neutrophil responses induced by ATP and PAF*. The correlation (*r*) was used to determine the direction (positive or negative) and quantify the strength of the association also between the ATP and PAF induced responses. The results obtained, when combining responses from the allosteric modulators in a scatterplot with a fitted linear regression curve as well as calulation of the Pearson coeficcients of the ATP and PAF responses (Fig 6A), revealed not only a strong positive correlation (r = 0.89) but also the linear correlation was strong (R^2^ 0.79; Fig 6A). The conclusions that could be drawn from the Bland-Altman graphs (Fig 6B), were that i) that PAF response was almost always higher than the ATP response and ii) that there was a positive trend.

*Direct correlation between the FFAR2 transduced neutrophil responses*. Comparing the responses for each allosteric modulator, by the more direct way to determine the similarities/differences between the effects of the allosteric FFAR2 modulators on the NADPH-oxidase activity, showed when comparing the PAF and ATP induced neutrophil response responses by the more direct way, that there were no differences in the ratio between the peak values of the PAF and ATP responses (Fig 6C). This suggests that the allosteric modulated FFAR2 was activated in a similar but not identical way by PAF and ATP.

#### 3.3.5. Relation between the neutrophil responses when FFAR2 was activated through receptor cross-talk by two Gα_i_ coupled GPCRs

**Fig 7:**
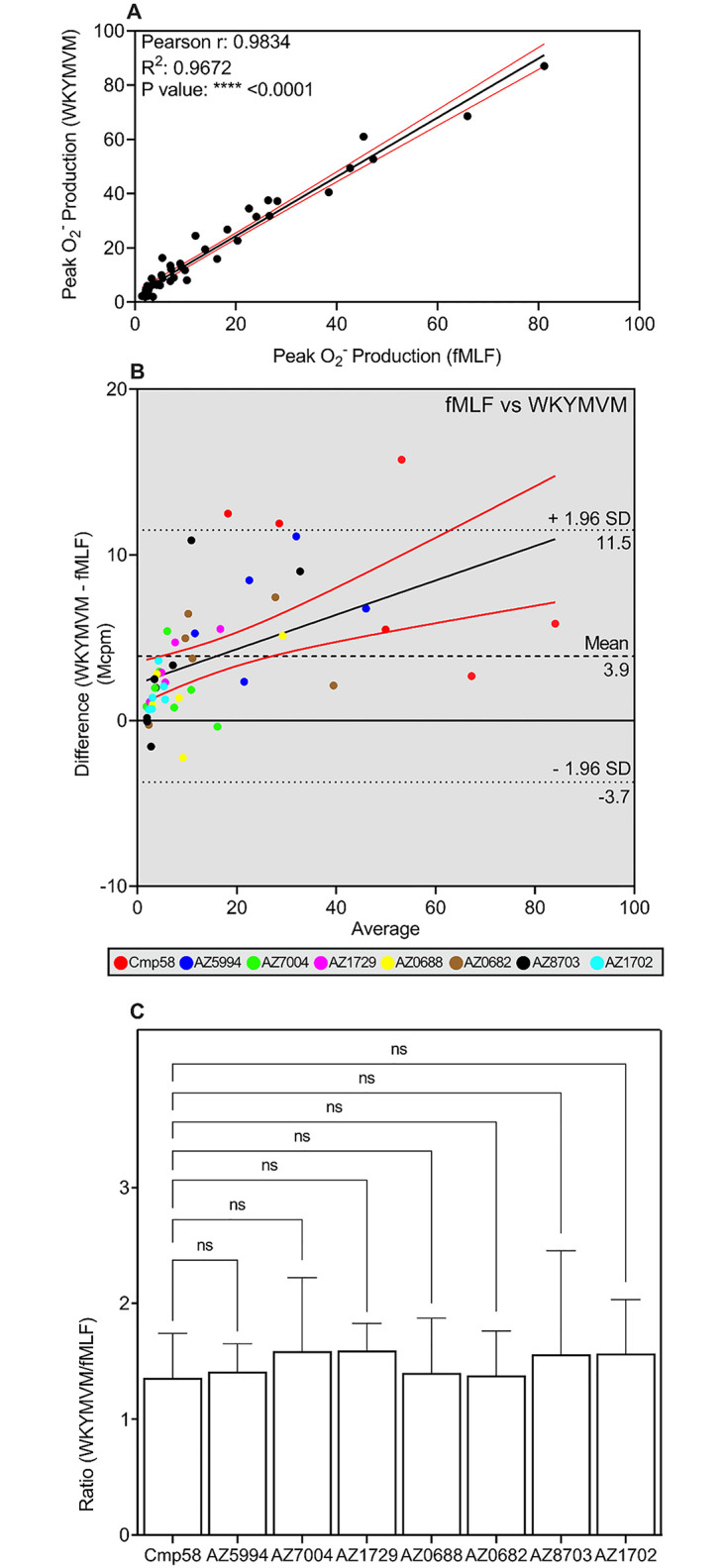
Activation by fMLF or WKYMVM of neutrophils sensitized with allosteric FFAR2 modulators. (**A**-**C**) Human neutrophils were sensitized for 5 min with allosteric FFAR2 modulators (1 μM each) and activated either by fMLF (0.5 nM) or WKYMVM (2.5 nM). O_2_^-^ production was measured continuously and the peak responses in neutrophils activated with fMLF and WKYMVM, respectively were determined for each allosteric modulator. Six independent experiments were performed with cells isolated from healthy donors. (**A**) Scatter plot for correlation between the peak values (O_2_^-^ production in relative light units; Mcpm) with fMLF (0.5 nM; abscissa) and WKYMVM (2.5 nM; ordinate) as the activating agonists. The responses obtained with all allosteric FFAR2 modulators were included. The black solid line corresponds to a linear regression fit and the red solid lines correspond to the 95% confidence slope for the linear regression fit. Pearson coefficients are also given. (**B**) Bland-Altman analysis of the peak values (O_2_^-^ production in relative light units; Mcpm) with fMLF and WKYMVM as the activating agonists. The responses obtained with all allosteric FFAR2 modulators were included and used to estimate the agreement between the readouts. The Bland-Altman plot for the differences (values for WKYMVM–values for fMLF in Mcpm) were plotted (ordinate) against (abscissa) the averages (peak values of (WKYMVM + fMLF)/2). Line of equality is presented (black dashed line), and the 95% upper and lower CI of limits of agreement are included (black dotted lines). Linear regression line of the differences is drawn as a black line that is combined with the 95% confidence interval (red lines above and below the linear regression line. The responses induced by WKYMVM and fMLF, respectively, have been color-coded with a color that is unique for each allosteric FFAR2 modulator included in the Bland-Altman plot. (**C**) The ratio for each allosteric FFAR2 modulator, between the WKYMVM and fMLF responses (O_2_^-^ production in relative light units; Mcpm) were calculated and presented in a bar graph. One-way ANOVA followed by Dunnett’s post-hoc test was used to calculate significant difference deviation from the ratio obtained with Cmp58 as the amplifying allosteric FFAR2 modulator. Six independent experiments with cells isolated from healthy donors were performed and the results are given as mean +SD.

We have earlier shown that Low (normally non-activating) concentrations of agonists (fMLF and WKYMVM) recognized by formyl peptide 1 and 2, respectively [35, 36], activate the O_2_^-^ producing NADPH-oxidase in the presence of an allosteric FFAR2 modulator [10, 19]. Also, this transactivation is inhibited by FFAR2 specific antagonists, but in contrast to the ATP and PAF induced activation, the Gα_q_ inhibitor (YM-254890) has no effect on FPR signaling. This is in agreement with the generally accepted opinion that the FPRs couple to a Gα_i_ containing G protein [37]. The data obtained from the Pearson scatterplot, comparing peak responses induced fMLF and WKYMVM through the FFAR2 cross-talk mechanism, the positive linear correlation had an R^2^ value of 0.97 (Fig 7A). The agreement between fMLF and WKYMVM was obvious also when plotting the Bland-Altman scatter plots of the two peptides (Fig 7B). The bias for the agreement between WKYMVM and fMLF is 3.9 Mcpm, which consider as an acceptable level of differentiation [38] between two different modes of activation. However, the responses induced by fMLF and WKYMVM were low for the majority of the allosteric modulators (Fig 7A).

*Direct correlation between the FFAR2 transduced neutrophil responses induced by FPR agonists*. We performed a comparison of the responses for each allosteric FFAR2 modulator as a ratio to enable a more direct way to determine the similarities/differences between the effects of the allosteric FFAR2 modulators on the NADPH-oxidase activity (Fig 7C). The responses induced by low concentrations of fMLF and WKYMVM had no significant ratio difference between the peak values of the WKYMVM and fMLF, implying that similar downstream signals that transactivated FFAR2 were generated by both FPR1 and FPR2.

## Discussion

Activation of neutrophils by various agonists recognized by GPCRs such as the free fatty acid receptors (FFARs) and formyl peptide receptors (FPRs) include receptor downstream signaling events such a transient rise in the cytosolic concentration of calcium ions and recruitment of β-arrestin as well as cell functions such as granule secretion, chemotactic migration and production of reactive oxygen species (for details see [13]. Receptor activities may be regulated not only by agonists but also by receptor specific allosteric modulators [39], and we show that allosteric modulators specific for free fatty acid receptor 2 (FFAR2) transfer not only the orthosteric FFAR2 agonist propionate to a neutrophil activating ligand, but also the responses induced by agonists for P2Y_2_R (receptor for ATP), PAFR (receptor for PAF) and the FPRs (FPR1, receptor for fMLF; FPR2, receptor for WKYMVM) were positively modulated. All receptors belong to the family of GPCRs. In addition to the G protein signaling cascade that is initiated by agonist binding and a structural change of the cytoplasmic GPCR domains, the allosterically modulated FFAR2 may also be activated by receptor cross-talk/transactivation mechanisms. This mode of activation of FFAR2 is initiated down-stream of P2Y_2_R, PAFR and the FPRs, by signals generated by these receptors on the inside of the plasma membrane. FFAR2 has been shown to have two distinctly different allosteric receptor sites [9–11, 23, 31], and allosteric modulators that selectively bind to one or the other of the two allosteric sites have been used to investigate the link (if any) between activation of FFAR2 by the classical out-side-in signaling mechanism and the intracellular receptor cross-talk signaling mechanism. The data presented herein support the hypothesis that the mechanism by which the neutrophil NADPH-oxidase is activated by FFAR2 differs when achieved through agonist binding to the orthosteric site, and when achieved by signals generated down-stream of a cross-talking/transactivating neutrophil receptor.

Irrespectively of the precise mechanism by which allosteric modulators works [40, 41], allosteric GPCR modulation should by definition solely affect receptor-mediated responses induced by agonists that are recognized by the modulated receptor. However, the data presented suggests this restriction for allosteric modulators is not valid for FFAR2. The activation of the neutrophil NADPH-oxidase is induced by ATP, PAF as well as low nanomolar concentrations of FPR agonists, all depends on an allosterically modulated FFAR2. This is evident from the fact that FFAR2 specific antagonists inhibit this receptor cross-talk response and no allosteric receptor cross-talk activation is achieved in cells lacking FFAR2 [10, 11, 19]. The precise signaling mechanism by which the neutrophil GPCRs (i.e., P2Y_2_R, PAFR, FPR1 and FPR2) activate the allosterically modulated FFAR2 has not been disclosed, but the fact that the receptor cross-talk activating signal(s) generated by P2Y_2_R and PAFR require a functional Gα_q_ subunit, suggests that the receptor cross-talk signals are generated down-stream of the G protein and activate FFAR2 from the cytosolic side of the plasma membrane. Although the FPRs probably couple to a Gα_i_ containing G protein [37], similar postulations have earlier been made regarding the FPR1 and FPR2 receptor cross-talk transactivation signal(s).

The modulators used in the study bind to different allosteric sites [9, 11], affect the FFAR2 response induced by propionate to varying degrees, and were used to determine the correlation between the two different FFAR2 activation mechanisms; that is outside-in activation induced by the orthosteric FFAR2 agonist and inside-in receptor cross-talk FFAR2 activation.

The data presented showed that there was a moderate positive overall correlation between the propionate and ATP induced responses when all allosteric modulators were included. The correlation/agreement was determined from Pearson and Bland-Altman plots, respectively. Generally Bland-Altman plots are used to show the agreement between two activation processes but unfortunately, there are no values for limits of agreement that are universal in indicating whether an agreement is good or bad [38]. This means that there is no predefined cut-off value to refer to, which means experimental context is crucial. The lack of a robust correlation between the propionate and ATP responses was apparent when the ratios between the two responses were determined separately for the different allosteric modulators. Whereas the ratios between the propionate and ATP induced responses were around one for the two “parent” allosteric FFAR2 modulators (i.e., Cmp58 and AZ1729), the values were significantly lower for the other modulators with AZ8703 and AZ1702 being the lower extremes. These data suggest that the allosterically modulated FFAR2 can adopt conformations that differ in the ability to transduce the signals induced by an orthosteric agonist and the signals generated down-stream of P2Y_2_R, respectively.

The results obtained when PAF was used to activate neutrophils, show that a receptor cross-talk/transactivation of FFAR2 was obtained, with similar characteristics as that induced by ATP. These data suggest that the allosterically modulated FFAR2 can adopt a conformation that is better suited to transduce the signals induced by an orthosteric agonist and the signals generated down-stream of the PAFR. In contrast to the non-activating P2Y_2_R agonist ATP, PAF is a weak activating agonist also in the absence of an FFAR2 modulator, but the fact that the cross-talk activation of FFAR2, induced both by PAF and ATP, was inhibited by a Gα_q_ selective inhibitor, suggests that similar (but not identical) signals are generated down-stream of the PAFR and the P2Y_2_R. Accordingly, the correlation/agreement determined from Pearson and Bland-Altman plots is in line with this similarity. Generally, the PAF induced response is more pronounced than the ATP response (a mean bias in Bland-Altman of 16.4), but the similarity was apparent when the ratios between the two responses were determined separately for the different allosteric modulators. No significant differences in the ratios between the PAF and ATP induced responses were identified, suggesting that similar FFAR2 signals are generated by the two receptors.

We have earlier shown that provided that FFAR2 is allosterically modulated, a response is induced by FPR specific agonists at low (non-activating) concentrations. The FPRs being Gα_i_ coupled (for details see [13]), suggest that the receptor cross-talk/transactivation signals may also be generated downstream of both Gα_i_ containing G proteins. The two FPRs share similar signaling/activation patterns [36, 42] and in accordance with this the ratios between the responses induced by the two agonists were close to one for all allosteric modulators. These data suggest that identical intracellular cross-talk signals were generated by these receptors, signals that activate the allosterically modulated FFAR2.

The two allosteric binding sites of FFAR2 have not yet been identified [9], but according to the binding sites model, allosteric modulator that bind to the same site as Cmp58 (AZ5994 and AZ7004 being “Cmp58-like”) and AZ1729 (AZ0688, AZ0682, AZ8703 and AZ1702 being “AZ1729-like”), should induce the same response pattern as that for the respective parent compound. This was, however, not what was found; this is illustrated by the fact that the response induced by ATP in AZ7004 sensitized neutrophils was fairly low compared to the response induced by propionate, whereas no such difference was evident for AZ5994. AZ7004 is the *S*-enantiomer of 4CMTB (also known as AMG7703), an earlier described racemic phenylacetamide, classified as an allosteric FFAR2 agonist. Despite the fact that the structure of AZ7004 is very similar to that of AZ5994, the response pattern following sensitization with the latter follow that of the parent compound Cmp58. The same response pattern as that seen with AZ1729 should be obtained with different “AZ1729-like” allosteric modulators. Once again this was not what was found; whereas the response patterns induced by ATP and PAF in AZ0688 and AZ0682 sensitized neutrophils were very similar to that with the parent compound AZ1729, no such similarity was evident for AZ8703 and AZ1702.

**Fig 8:**
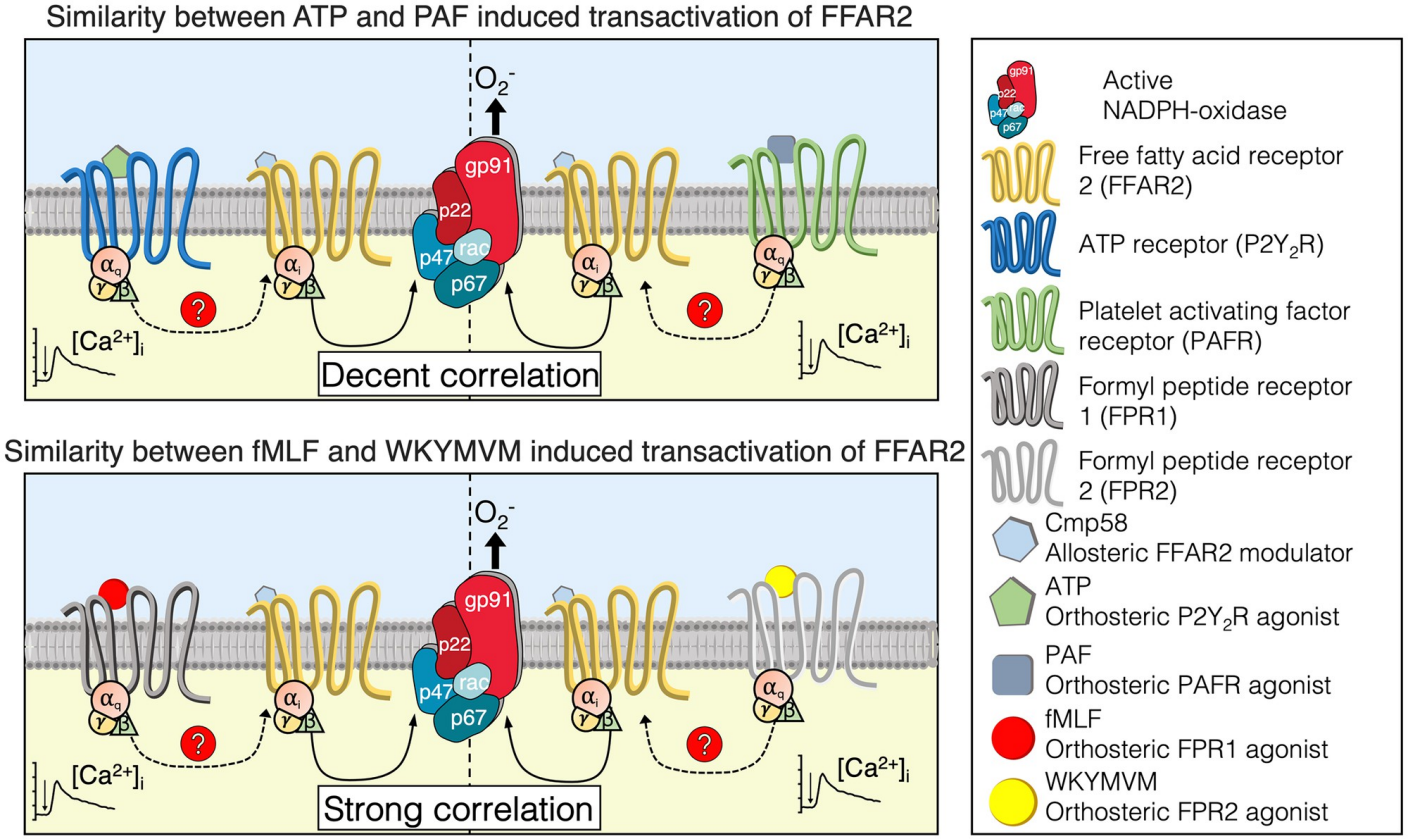
**Top panel: Model for PAF and ATP induced transactivation of the allosterically modulated FFAR2.** According to the data presented in the study, the allosterically modulated FFAR2 is activated by signals generated by the ATP (receptor to the far left) and PAF (receptor to the far right) receptors. The transactivation signals are generated down-stream (on the cytosolic side) of the Gα_q_ containing G protein coupled to P2Y_2_R (the ATP receptor) and PAFR (receptor for PAF), respectively, and the signals activate FFAR2 provided that this receptor is allosterically modulated. The aim was to determine if the responses induced by transactivating receptors were directly correlated; if so, the hypothesis is that the two receptors generate the same signal that ultimately transactivate FFAR2. The similarity between the transactivation responses was apparent when the ratios between the two responses were determined separately for the different allosteric modulators, suggesting that similar (but maybe not identical) FFAR2 transactivation signals are generated by the PAF and ATP receptors. **Bottom panel: Model for fMLF (FPR1 agonist) and WKYMVM (FPR2 agonist) induced transactivation of the allosterically modulated FFAR.** According to the data presented in the study, the allosterically modulated FFAR2 is activated by signals generated by the FPRs. The transactivation signals are hypothetically generated down-stream (on the cytosolic side) of the Gα_i_ containing G protein coupled to the FPRs and the signals activate FFAR2 provided that this receptor is allosterically modulated. The aim was to determine if the responses induced also by these transactivating receptors were directly correlated; if so, the hypothesis is that the two receptors generate the same signal that ultimately transactivate FFAR2. The similarity between the transactivation responses was strongly correlated when the ratios between the two responses were determined separately for the different allosteric modulators, suggesting that similar (possibly identically FFAR2 transactivation signals are generated by the two FPRs.

The proposed signaling mechanism by which the cross-talking receptors for ATP, PAF, fMLF, and WKYMVM activates allosterically modulated FFAR2 (see Fig 1), starts with an activation of a G protein down-stream of P2Y_2_R, PAFR, FPR1/FPR2 and the signal(s) generated then activates FFAR2 from the cytosolic side of the membrane, and the receptor crosstalk/transactivation signals may be generated down-stream of both Gα_i_ and Gα_q_ containing G proteins. These data suggest similar but possibly not identical signals are generated downstream the different G proteins (Fig 8), but the precise nature of these receptor crosstalk/transactivation signals remains to be determined.

Taken together, our data showing that allosteric FFAR2 modulators affect not only the activating potential of propionate, the natural (orthosteric) agonist of the modulated receptor, but also that of several other inflammatory mediators, raises question not only about the regulatory roles of FFAR2 in inflammatory settings, but also the mechanisms by which neutrophil GPCRs communicate to activate the ROS generating NADPH-oxidase. Our working hypothesis is that a receptor down-stream signals generated by several other neutrophil GPCRs activate the allosterically modulated FFAR2. The signal has not been identified we but we can exclude an allosteric modulator dependent increased phosphorylation of one of the cytosolic NADPH-oxidase components (p47^phox^) as the molecular mechanism. This has been described as a neutrophil priming mechanism [43], but if this was the mechanism, an activation would be obtained also if the order was reversed, by which the allosteric modulator and the transactivating agonist was added to the cells. reversed; this was, however, not the case. In our opinion, a transient rise in the cytosolic concentration of calcium ions induced by the transactivating receptors is a more attractive hypothesis that will be investigated in our future work. In addition, the activation characteristics of allosterically modulated FFAR2s depend not only on the involvement of one or the other the two different allosteric receptor sites, but also on the nature of the allosteric modulator. Accordingly, the model system with FFAR2 together with the different transactivating neutrophil receptors, will be useful in future studies of the allosteric modulation phenomenon and to increase our understanding about the biology of GPCRs biology and the potential of these receptors a drug target to regulate inflammation.

## Abbreviations

AZ1729: an FFAR2 modulator
CATPB: (*S*)-3-(2-(3-chlorophenyl)acetamido)-4-(4-(trifluoromethyl)phenyl)butanoic acid an FFAR2 antagonist/inverse agonist
CL: chemiluminescence
Cmp1: (*R*)-3-benzyl-4-(cyclopropyl(4-(2,5-dichlorophenyl)thiazol-2-yl)amino)-4-oxobutanoic acid a small molecule orthosteric FFAR2 agonist
Cmp58: an allosteric FFAR2 modulator
FFAR2: free fatty acid receptor 2
GPCR: G-protein coupled receptor
HRP: horse radish peroxidase
KRG: Krebs-Ringer Glucose phosphate buffer
ns: significant difference
ROS: reactive oxygen species
TNF-α: tumor necrosis factor α
